# Cardiomyocyte-specific deletion of GCN5L1 in mice restricts mitochondrial protein hyperacetylation in response to a high fat diet

**DOI:** 10.1038/s41598-020-67812-x

**Published:** 2020-06-30

**Authors:** Dharendra Thapa, Janet R. Manning, Michael W. Stoner, Manling Zhang, Bingxian Xie, Iain Scott

**Affiliations:** 1 Vascular Medicine Institute, University of Pittsburgh, Pittsburgh, PA 15261 USA; 2 Center for Metabolism, University of Pittsburgh, Pittsburgh, PA 15261 USA; 3 Division of Cardiology, Department of Medicine, University of Pittsburgh, BST E1256, 200 Lothrop Street, Pittsburgh, PA 15261 USA

**Keywords:** Physiology, Metabolism, Mitochondria

## Abstract

Mitochondrial lysine acetylation regulates several metabolic pathways in cardiac cells. The current study investigated whether GCN5L1-mediated lysine acetylation regulates cardiac mitochondrial metabolic proteins in response to a high fat diet (HFD). GCN5L1 cardiac-specific knockout (cKO) mice showed significantly reduced mitochondrial protein acetylation following a HFD relative to wildtype (WT) mice. GCN5L1 cKO mice did not display any decrease in ex vivo cardiac workload in response to a HFD. In contrast, ex vivo cardiac function in HFD-fed WT mice dropped ~ 50% relative to low fat diet (LFD) fed controls. The acetylation status of electron transport chain Complex I protein NDUFB8 was significantly increased in WT mice fed a HFD, but remained unchanged in GCN5L1 cKO mice relative to LFD controls. Finally, we observed that inhibitory acetylation of superoxide dismutase 2 (SOD2) at K122 was increased in WT (but not cKO mice) on a HFD. This correlated with significantly increased cardiac lipid peroxidation in HFD-fed WT mice relative to GCN5L1 cKO animals under the same conditions. We conclude that increased GCN5L1 expression in response to a HFD promotes increased lysine acetylation, and that this may play a role in the development of reactive oxygen species (ROS) damage caused by nutrient excess.

## Introduction

Growing evidence points to a crucial role played by lysine acetylation, a reversible post-translational modification, in the regulation of mitochondrial bioenergetics and metabolism in the heart. We and several others have shown that mitochondrial protein acetylation is significantly increased in various tissues during obesity and diabetes^1–4^. This correlates with an increase in the abundance of GCN5L1, a mitochondrial acetyltransferase protein that has previously been shown to counter the activity of the mitochondrial deacetylase enzyme SIRT3^5^. In several tissues, GCN5L1 has been shown to regulate the acetylation of mitochondrial fatty acid oxidation (e.g. LCAD, HADHA, SCAD), glucose oxidation (e.g. PDH), and electron transport chain (ETC) proteins (e.g. NDUFA9 from complex I)^3–5^. In cultured cardiac cells, loss of GCN5L1 has been shown to limit mitochondrial respiratory capacity under uncoupled conditions^6^, reduces fatty acid use for both ATP production and cellular proliferation^4^, and results in the uncoupling of glycolysis from glucose oxidation for energy production^7^. While the effects of GCN5L1 depletion on mitochondrial metabolism have been studied in cardiac cells in vitro, it is not known whether this regulation is operable in vivo, or whether changes in nutrient conditions affect functional outputs.

In the present study, we examined the effect of a long-term high fat diet (HFD) in wildtype (WT) and cardiac-specific GCN5L1 knockout (cKO) mice. Despite similar levels of cardiac hypertrophy, GCN5L1 cKO mice maintained cardiac workload ex vivo after HFD exposure, while WT mice decreased output by ~ 50% under the same conditions. GCN5L1 expression is necessary for the acetylation of the ETC Complex I protein NDUFB8, as well as inhibitory acetylation of mitochondrial superoxide dismutase 2 (SOD2) at K122 under HFD conditions. This results in decreased lipid peroxidation in GCN5L1 cKO mice, relative to wildtype animals, following exposure to a HFD. Taken together, these data indicate that the acetylation of cardiac mitochondrial proteins by GCN5L1 under HFD conditions may lead to cardiac dysfunction, as a downstream consequence of increased cellular damage from lipid peroxidation.

## Results

### Loss of GCN5L1 decreases mitochondrial protein acetylation under HFD conditions

**Figure 1:**
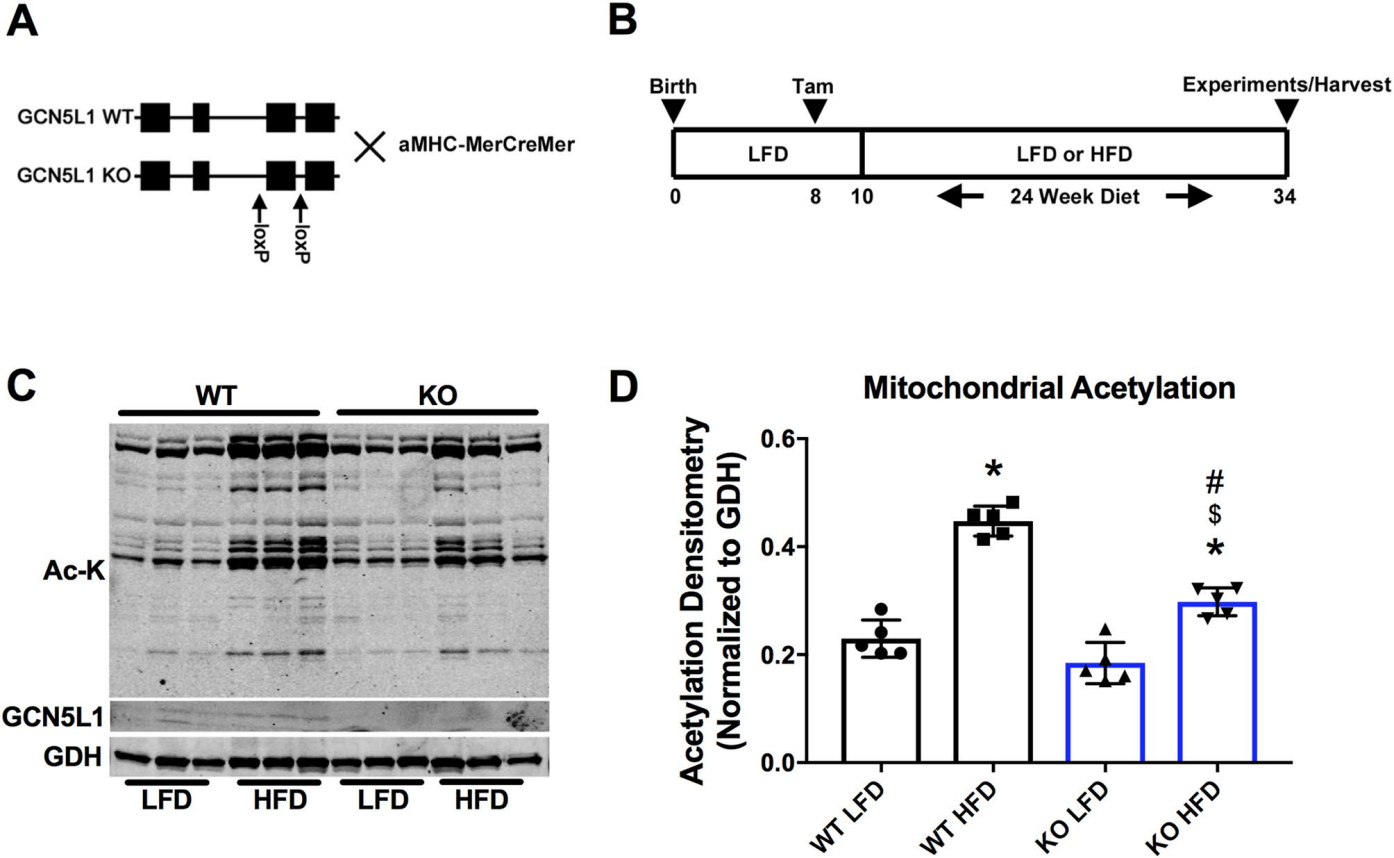
Characterization of mitochondrial acetylation in WT and GCN5L1 cKO animals. (**A**) Genotype and breeding scheme of WT and GCN5L1 cKO transgenic mice. (**B**) Diet and tamoxifen (Tam) injection schedule of WT and GCN5L1 cKO mice. (**C**, **D**) Overall acetylation of mitochondria isolated from cardiac tissues was greatly increased in HFD WT animals, which was significantly attenuated in HFD cKO mice. Values are expressed as means ± SD, n = 5, *P < 0.05 versus WT LFD, ^$^*P* < 0.05 versus cKO LFD, ^#^*P* < 0.05 versus WT HFD.

We first examined the response of WT and GCN5L1 cKO mice to varying nutrient conditions, and placed groups of animals on a LFD or HFD for 24 weeks (Fig. 1A, B). We previously reported that increased acetylation of several mitochondrial metabolism enzymes, in response to nutrient excess, is regulated in part by GCN5L1 in vitro^4^. To investigate a role for GCN5L1 in this process in vivo, we first examined the total level of mitochondrial protein acetylation in WT and GCN5L1 cKO mouse hearts under LFD and HFD. WT HFD mice displayed a ~ twofold increase in mitochondrial acetylation relative to WT LFD mice (Fig. 1C, D). While still significantly elevated relative to LFD mice, there was a significant ~ 67% decrease in mitochondrial acetylation in GCN5L1 cKO HFD mice relative to their WT HFD controls (Fig. 1C, D). This was not due to changes in the expression or acetylation status of the deacetylase enzyme SIRT3, which were not different between WT and cKO mice (Supplemental Fig. 3). We therefore conclude that loss of GCN5L1 limits the accumulation of hyperacetylated mitochondrial proteins in response to nutrient excess.

### Mice with cardiomyocyte-specific deletion of GCN5L1 do not display decreased ex vivo cardiac workload in response to a HFD

**Figure 2:**
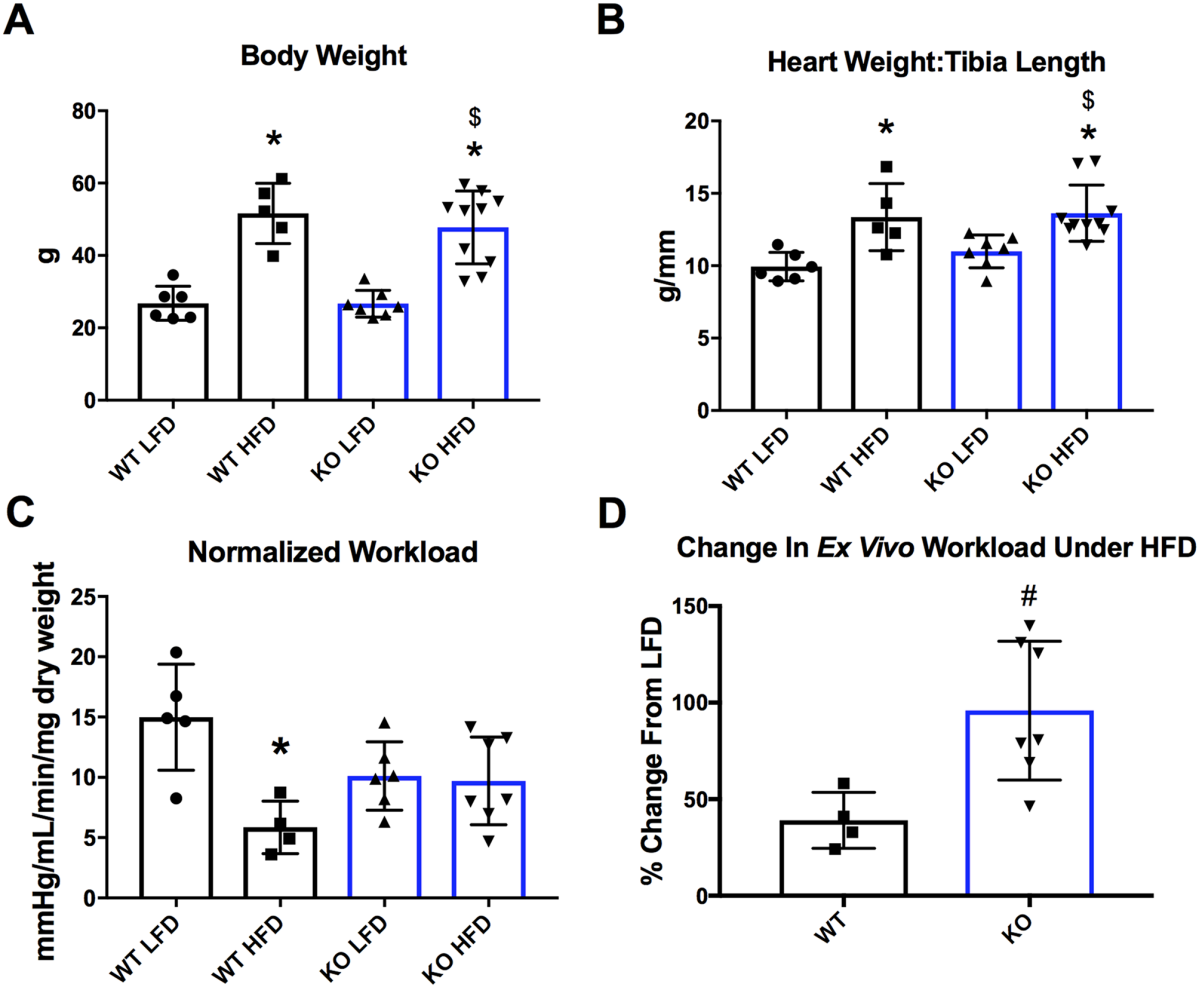
Impact of GCN5L1 deletion on physical features and cardiac workload ex vivo. (**A**, **B**) Exposure to a HFD led to similar significant increases in total body weight and heart weight: tibia length ratio in both WT and GCN5L1 cKO mice. (**C**) There was a significant, ~ 50% decrease in normalized ex vivo workload in WT HFD animals relative to WT LFD mice. This relative decrease in normalized ex vivo workload was not observed in GCN5L1 cKO mice. Values are expressed as means ± SD, n = 4–7, *P < 0.05 versus WT LFD, ^$^*P* < 0.05 versus cKO LFD, ^#^*P* < 0.05 versus WT HFD.

We next examined whether deletion of GCN5L1 in cardiomyocytes would affect cardiac anatomical and functional features in response to nutrient excess. Both HFD-fed WT and GCN5L1 cKO mice displayed significant elevations in body weight and heart weight:tibia length ratio relative to LFD controls, however there were no differences between the two genotypes (Fig. 2A, B). Under LFD conditions, GCN5L1 cKO mice displayed a minor decrease in ex vivo cardiac workload relative to LFD-fed WT mice, however this did not reach statistical significance (Fig. 2C). Under HFD conditions, WT mice displayed a significantly reduced (~ 50%) ex vivo cardiac workload compared to WT LFD controls, while no change was observed in GCN5L1 cKO mice between low fat and high fat conditions (Fig. 2C, D). We therefore conclude that loss of GCN5L1 cKO in the heart allows mice to maintain cardiac output under excess nutrient conditions.

### Role of GCN5L1 in electron transport chain (ETC) complex acetylation and redox status

**Figure 3:**
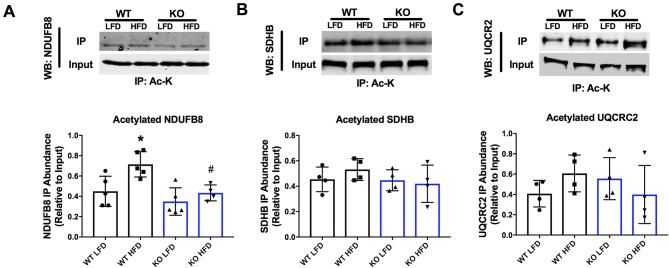
Acetylation status of mitochondrial electron transport chain proteins in response to a HFD. (**A**) Acetylated lysine immunoprecipitation from whole cardiac tissue showed an increase in the acetylation of NDUFB8 (Complex I) in WT HFD animals relative to GCN5L1 cKO mice under the same conditions. (**B**, **C**) There were no changes observed in the acetylation status of SDHB (Complex II) and UQCR2 (Complex III). Values are expressed as means ± SD, n = 4–5, *P < 0.05 versus WT LFD, ^#^*P* < 0.05 versus WT HFD.

We next investigated whether acetylation-driven changes in mitochondrial bioenergetics proteins may account for the loss of cardiac function in WT mice under HFD conditions. Several groups have reported that the acetylation of mitochondrial electron transport chain (ETC) enzymes inhibits their activity^5,9–11^, and we therefore examined the role of GCN5L1 in the acetylation of ETC complex proteins. Immunoprecipitation of proteins from whole cardiac tissue lysates with an acetylated lysine antibody demonstrated that there was a significant increase in the acetylation of Complex I subunit NADH dehydrogenase [ubiquinone] 1 beta subcomplex subunit 8 (NDUFB8) in GCN5L1 WT HFD animals, which was completely attenuated in GCN5L1 cKO mice (Fig. 3A). No significant changes were observed in acetylation of Complex II and III subunits SDHB and UQCR2, respectively (Fig. 3B, C). We therefore conclude that GCN5L1 promotes the acetylation of a Complex I subunit in response to nutrient excess, and that this change is abrogated by cardiomyocyte-specific deletion of this protein.

### Deletion of GCN5L1 in the heart limits SOD2 inhibitory acetylation and lipid peroxidation

**Figure 4:**
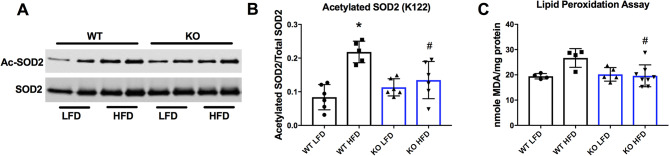
Impact of GCN5L1 deletion on ROS regulation and damage in response to a HFD. (**A**, **B**) Acetylation of superoxide dismutase 2 (SOD2) at K122 is significantly increased in whole cardiac tissue from WT animals in response to a HFD. There was no increase in SOD2 acetylation in GCN5L1 cKO mice under the same dietary conditions. (**C**) Cardiac lipid peroxidation in samples obtained from while cardiac tissue, a measure of ROS damage, is significantly lower in GCN5L1 cKO mice relative to WT mice under HFD conditions. Values are expressed as means ± SD, n = 4–8, *P < 0.05 versus WT LFD, ^#^*P* < 0.05 versus WT HFD.

ETC Complex I is a major source of reactive oxygen species production^12^, and loss of Complex I activity has been linked to decreased cardiac function in response to stress^13^. We next investigated whether changes in the acetylation status of Complex I proteins impacted the redox milieu in GCN5L1 cKO mice. Acetylation of mitochondrial superoxide dismutase 2 (SOD2) at lysine 122 (K122) has been shown to inhibit its enzymatic activity^14^. We observed a significant increase in the acetylation status of SOD2 at K122 in WT mice in response to a HFD, and this was significantly reduced in GCN5L1 cKO animals under the same conditions (Fig. 4A, B). Elevated SOD2 acetylation in WT mice on a HFD correlated with a moderate, yet non-significant increase in lipid peroxidation in WT animals (19.44 ± 1.06 versus. 26.68 ± 3.71, *P* = 0.09). This increase was significantly attenuated in GCN5L1 cKO HFD mice relative to WT HFD mice under the same dietary conditions (Fig. 4C). We therefore conclude that acetylation of SOD2 at K122 is regulated in part by GCN5L1, and that loss of this modification in GCN5L1 cKO animals results in decreased lipid peroxidation in response to HFD exposure.

## Discussion

Using a HFD feeding model combined with cardiac-specific deletion of GCN5L1, we demonstrate for the first time in vivo that GCN5L1 mediates the acetylation of NDUFB8 (ETC Complex I) and SOD2 (ROS amelioration) in response to nutrient excess. Furthermore, we show that decreased acetylation of these enzymes in GCN5L1 cKO mice correlates with decreased lipid peroxidation under HFD conditions. Finally, we show that a significant decrease in ex vivo cardiac workload found in WT animals under HFD conditions is not observed in GCN5L1 cKO mice. In summary, we conclude that GCN5L1 deletion limits the decline in cardiac function normally observed in mice under conditions of chronic nutrient excess.

We have previously shown that prolonged high fat feeding leads to the increased acetylation of mitochondrial proteins, which was in part regulated by GCN5L1 when tested under in vitro cell culture conditions^4^. To substantiate the role played by GCN5L1 in regulating the mitochondrial acetylome following exposure to a HFD, we utilized a newly described cardiac-specific GCN5L1 cKO animal model^20^. As anticipated, we observed a significant decrease in mitochondrial protein acetylation in GCN5L1 cKO animals under nutrient excess conditions. A number of studies have extensively reported the role of SIRT3-mediated mitochondrial protein deacetylation^2,15,16^, however the role of GCN5L1 in this context is much less known. Upon further investigation of specific proteins targeted by GCN5L1, we found that enzymes involved in the mitochondrial ETC and ROS protection pathways were substrates of GCN5L1-mediated protein acetylation.

It was previously reported that ETC Complex I is one of the major sites of ROS production in mitochondria^17–19^. Complex I deficiency has also been shown to increase mitochondrial protein acetylation and accelerate heart failure, via SIRT3-mediated hyperacetylation^13^. Furthermore, in a streptozotocin-induced type 1 diabetic model, enhanced mitochondrial protein lysine acetylation has been reported as a common consequence of increased fatty acid oxidation and Complex I defects^19^, which could ultimately be responsible for metabolic inflexibility of the diabetic heart. These authors reported no change in the mitochondrial deacetylase SIRT3, while the role of GCN5L1 was not examined^20^. We report for the first time that mitochondrial GCN5L1 partly regulates the acetylation of the Complex I protein subunit NDUFB8, which may contribute to complex activity defects under HFD conditions.

Superoxide dismutase 2 (SOD2) is the primary mitochondrial ROS scavenger that converts superoxide (a by-product of Complex I activity) to hydrogen peroxide, which is then ultimately converted to water by catalase and other peroxidases^21^. It has been reported that SIRT3-mediated deacetylation of SOD2 at K122 results in its increased enzymatic activity^14^. As such, we examined if the mitochondrial acetyltransferase activity of GCN5L1 regulated this acetylation site in our study, and found that deletion of GCN5L1 resulted in significantly decreased acetylation of SOD2 at K122 in cKO HFD mice relative to WT animals (Fig. 4A, B). This may contribute to the significantly decreased lipid peroxidation observed in GCN5L1 cKO HFD hearts (Fig. 4C). This stands in partial contrast to previous reports, which showed that GCN5L1 cKO hearts displayed increased protein carbonylation (a different marker of ROS damage) in response to ex vivo ischemic stress^8^. However, differences in the experimental model (acute ischemia versus. chronic HFD), age of mice (8–10 weeks versus. 34 weeks, respectively), and ROS damage proxy measurements (protein carbonylation versus. lipid peroxidation, respectively) make simple comparisons between these reports challenging. Work is currently underway to elucidate the functional outcome of K122 acetylation on SOD2 activity in response to acute (ischemia–reperfusion) versus chronic (HFD) derived ROS in the heart. It is expected that delineation of the response to ROS from these two etiologies may explain the differences observed between the previous and current studies, and clarify the role of GCN5L1 under each condition.

In summary, we report a novel role for GCN5L1 in the acetylation of ETC and ROS regulatory proteins in the heart, and show that deletion of GCN5L1 mitigates HFD-driven reductions in cardiac function ex vivo. These findings suggest that GCN5L1 may be an important regulator of cardiac function, and further in vivo studies in this field are merited.

## Materials and methods

### Animal care and use

Animals were housed in the University of Pittsburgh animal facility under standard conditions with ad libitum access to water and food, and maintained on a constant 12 h light/12 h dark cycle. Male GCN5L1 WT and cKO animals were fed either a standard low fat diet (LFD; 70% carbohydrate, 20% protein, 10% fat; Research Diets D12450B), or a high fat diet (HFD; 20% carbohydrate, 20% protein, 60% fat; Research Diets D12492), for 24 weeks. At the end of 24 weeks, animals were euthanized and heart tissues excised for analysis. Experiments were conducted in compliance with National Institutes of Health guidelines, and followed procedures approved by the University of Pittsburgh Institutional Animal Care and Use Committee.

### Transgenic mice

Cardiomyocyte-specific inducible GCN5L1 knockout mice used in the studies were generated as previously reported^8^. Briefly, mice were generated on a C57BL/6J background by crossing αMHC-Cre (Jax B6.FVB(129)-Tg(Myh6-cre/Esr1*)1JMK/J) mice (i.e. αMHC-MerCreMer) to mice with LoxP sites introduced around exon 3 of Bloc1s1 (GCN5L1^Flox^). Cardiomyocyte-specific GCN5L1 deletion was induced via tamoxifen injection (single 40 mg/kg IP injection) and confirmed by qPCR (data not shown) and immunoblot (see Supplemental Fig. 1).

### Protein isolation

For whole heart protein lysate, tissues were minced and lysed in CHAPS buffer (1% CHAPS, 150 mM NaCl, 10 mM HEPES, pH 7.4) on ice for ~ 2 h. Homogenates were spun at 10,000*g*, and supernatants collected for western blotting or co-immunoprecipitation experiments. Mitochondria isolation was performed using the Qproteome Mitochondria isolation kit (Qiagen, catalog number 37612). Briefly, heart tissues were cut and homogenized in the provided lysis buffer. After separating the cytoplasmic fraction by centrifuging at 1,000*g* at 4 °C for 10 min, the remaining pellet was suspended in disruption buffer and centrifuged at 1,000*g* at 4 °C for 10 min. The supernatant was retained and centrifuged at 6,000*g* at 4 °C for 10 min to obtain the mitochondrial pellet. Mitochondria were reconstituted in the appropriate buffer depending upon the experiments performed.

### Western blotting

Protein lysates were prepared in LDS sample buffer, separated using Bolt SDS/PAGE 4–12% or 12% Bis–Tris gels, and transferred to nitrocellulose membranes (all Life Technologies). Protein expression was analyzed using the following primary antibodies: rabbit acetyl-lysine (Ac-K, catalog number 9441), rabbit sirtuin 3 (SIRT3, catalog number 5490), and rabbit glutamate dehydrogenase (GDH, catalog number 12793) from Cell Signaling Technologies; rabbit acetylated SOD2 (K122, catalog number ab214675), rabbit SOD2 (catalog number ab13533), and mouse OXPHOS cocktail (to analyze NDUFB8, SDHB and UQCR2 protein content, catalog number ab110413) from Abcam. Fluorescent anti-mouse or anti-rabbit secondary antibodies (red, 700 nm; green, 800 nm) from Li-Cor were used to detect expression levels. Protein densitometry was measured using Image J software (National Institutes of Health, Bethesda, MD). The full membranes of cropped blots may be found in Supplemental Fig. 2.

### Co-immunoprecipitation

For co-immunoprecipitation experiments, protein lysates were harvested in CHAPS buffer, and equal amounts of protein were incubated overnight at 4 ºC with rabbit acetyl-lysine antibody (Ac-K; Cell Signaling). Immunocaptured proteins were isolated using Protein-G agarose beads (Cell Signaling Technology, catalog number 9007), washed multiple times with CHAPS buffer, and then eluted in LDS sample buffer (Life Technologies) at 95 °C. Samples were separated on 12% Bis–Tris Bolt gels and probed with appropriate antibodies. Protein densitometry was measured using Image J software (National Institutes of Health, Bethesda, MD).

### Isolated working heart

Cardiac ex vivo workload was calculated using a Harvard Apparatus ISHR isolated working heart system as previously described^8^. Hearts from anesthetized mice were rapidly excised and cannulated via the aorta in warm oxygenated Krebs–Henseleit buffer (118 mM NaCl, 25 mM NaHCO_3_, 0.5 mM Na-EDTA [disodium salt dihydrate], 5 mM KCl, 1.2 mM KH_2_PO_4_, 1.2 mM MgSO_4_, 2.5 mM CaCl_2_, 11 mM glucose). Retrograde (i.e. Langendorff) perfusion was initiated to blanch the heart, maintained at a constant aortic pressure of 50 mmHg with a peristaltic pump through a Starling resistor. A small incision was next made in the pulmonary artery to allow perfusate to drain, and the heart was paced at a rate slightly higher than endogenous (~ 360–500 bpm). The left atrium was then cannulated via the pulmonary vein, and anterograde perfusion was initiated with a constant atrial pressure of 11 mmHg against an aortic workload of 50 mmHg. Left ventricle pressure was measured via Mikro-tip pressure catheter (Millar) carefully inserted into the LV through the aorta. The work-performing heart was permitted to equilibrate for 30 min to establish baseline functional parameters.

Cardiac ex vivo workload was calculated as the difference between recorded atrial and aortic pressures, multiplied by the cardiac output flow parameter. Workload was then normalized for each heart by dry heart weight (determined by measuring the ratio of a small section of air-dried heart tissue to its wet weight, and multiplying the entire heart wet weight by that ratio), and expressed as mmHg/mL/min/g. The difference in workload between LFD and HFD was determined as the percent normalized workload of the HFD relative to LFD for each genotype.

### Biochemical assays

Lipid peroxidation was measured using a commercial kit (Sigma-Aldrich, catalog number MAK085) according to the manufacturer’s instructions.

### Statistics

Graphpad Prism software was used to perform statistical analyses. Means ± SD were calculated for all data sets. Data were analyzed using two-way ANOVA with Sidak post-hoc testing to determine differences between genotypes and feeding groups. Data were analyzed with two-tailed Student’s T-Tests to determine differences between single variable groups. Data were tested for normal distribution using the Kolmogorov–Smirnov test. *P* < 0.05 was considered statistically significant.

## Supplementary information

Supplementary information.
