# Supplementary material for: Cardiomyocyte-specific deletion of GCN5L1 in mice restricts mitochondrial protein hyperacetylation in response to a high fat diet
Source: Sci Rep. 2020 Jun 30;10:10665. doi: 10.1038/s41598-020-67812-x (PMC7326908; doi:10.1038/s41598-020-67812-x)
Supplement: Supplementary file 1 — Supplementary information. [file 41598_2020_67812_MOESM1_ESM.docx]

**Cardiomyocyte-Specific Deletion of GCN5L1 in Mice Restricts Mitochondrial Protein Hyperacetylation in Response to a High Fat Diet**

Dharendra Thapa, Janet R Manning, Michael W. Stoner, Manling Zhang, Bingxian Xie, and Iain Scott

Vascular Medicine Institute, Center for Metabolism, and Division of Cardiology, Department of Medicine, University of Pittsburgh, Pittsburgh, PA 15261

**SUPPLEMENTAL INFORMATION**

**Supplemental Figure 1**

**
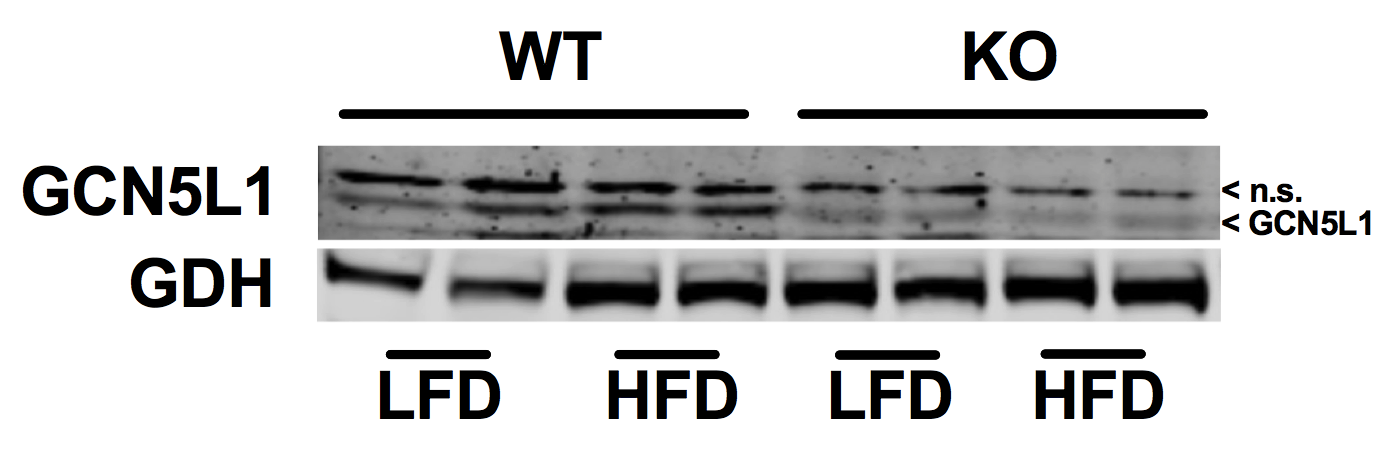
**

**Supplemental Figure 1. Confirmation of GCN5L1 depletion.** GCN5L1 expression in cKO mouse hearts was greatly depleted relative to WT hearts. As these lysates were obtained from whole cardiac tissue, a small level of GCN5L1 expression remains, as the MerCreMer does not delete GCN5L1 in non-cardiomyocyte cell types.

**Supplemental Figure 2**

**
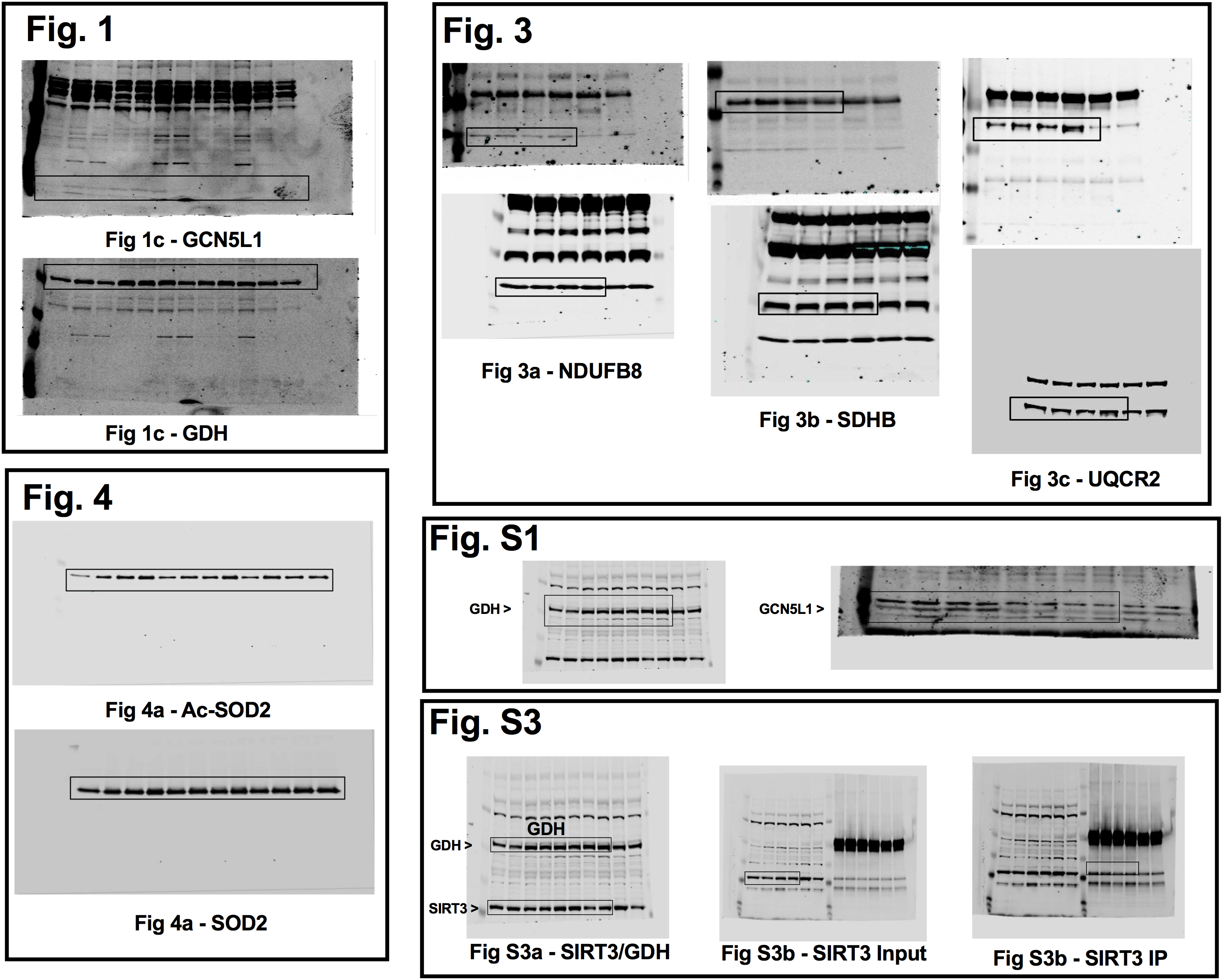
**

**Supplemental Figure 2. Full membranes from cropped images used in the main manuscript.** Note: The GCN5L1, SIRT3 and GDH blots used in Figures S1 and S3 were ­­­all obtained from the same membrane. As such, the same GDH loading control blot has been used in both.

**Supplemental Figure 3**

**
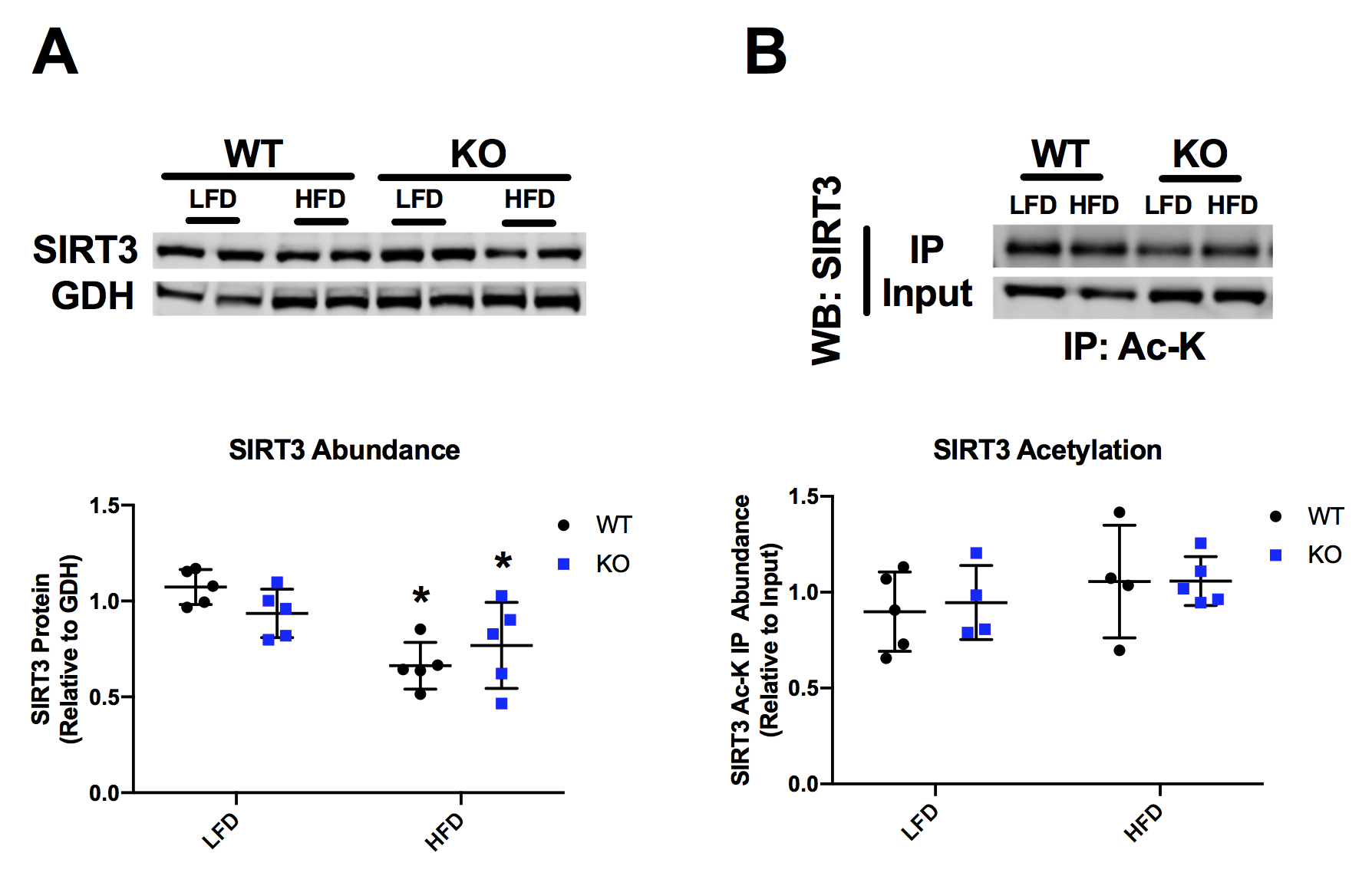
**

**Supplemental Figure 3. SIRT3 expression and acetylation level.** (A) SIRT3 was decreased in both HFD mouse groups, with no difference between WT and cKO cohorts. (B) There was no change in SIRT3 acetylation between WT and cKO mice under either diet condition.
